# Circulating insulin-like growth factors and Alzheimer disease: A mendelian randomization study

**DOI:** 10.1212/WNL.0000000000004854

**Published:** 2018-01-23

**Authors:** Dylan M. Williams, Ida K. Karlsson, Nancy L. Pedersen, Sara Hägg

**Affiliations:** From the Department of Medical Epidemiology & Biostatistics (D.M.W., I.K.K., N.L.P., S.H.), Karolinska Institutet, Stockholm, Sweden; and Department of Psychology (N.L.P.), University of Southern California, Los Angeles.

## Abstract

**Objective:**

To examine whether genetically predicted variation in circulating insulin-like growth factor 1 (IGF1) or its binding protein, IGFBP3, are associated with risk of Alzheimer disease (AD), using a mendelian randomization study design.

**Methods:**

We first examined disease risk by genotypes of 9 insulin-like growth factor (IGF)–related single nucleotide polymorphisms (SNPs) using published summary genome-wide association statistics from the International Genomics of Alzheimer's Project (IGAP; n = 17,008 cases; 37,154 controls). We then assessed whether any SNP-disease results replicated in an independent sample derived from the Swedish Twin Registry (n = 984 cases; 10,304 controls).

**Results:**

Meta-analyses of SNP-AD results did not suggest that variation in IGF1, IGFBP3, or the molar ratio of these affect AD risk. Only one SNP appeared to affect AD risk in IGAP data. This variant is located in the gene *FOXO3,* implicated in human longevity. In a meta-analysis of both IGAP and secondary data, the odds ratio of AD per *FOXO3* risk allele was 1.04 (95% confidence interval 1.01–1.08; *p* = 0.008).

**Conclusions:**

These findings suggest that circulating IGF1 and IGFBP3 are not important determinants of AD risk. *FOXO3* function may influence AD development via pathways that are independent of IGF signaling (i.e., pleiotropic actions).

The insulin-like growth factor (IGF) axis may have a role in cognitive decline and the etiology of Alzheimer disease (AD).^1^ Activity of the axis decreases markedly with age throughout adulthood, with circulating growth hormone/IGF1 very low in those aged over 60 years.^2^ IGF1 purportedly has neuroprotective effects in adult animals, promoting neuronal survival and reducing tau phosphorylation.^3,4^ Consistent with experimental findings, epidemiologic studies have found that individuals with AD, all-cause dementia, or cognitive decline have lower circulating IGF1 and its main binding protein (IGFBP3) than cognitively intact individuals,^5–9^ although some findings have been contradictory.^10–12^ Higher circulating IGF2 exposure could also have a role in delaying cognitive decline.^10^

Based on these past findings, circulating IGF1 has been proposed by some as a modifiable target for AD treatment or prevention to test in trials.^6^ It therefore remains to be clarified as to whether IGF concentrations in circulation are a causal factor in AD etiology. To investigate this further using a novel approach, we tested the hypothesis that risk of developing AD would be lower in individuals with genetically predicted, long-term increases in exposure to circulating IGF1 and IGFBP3.

## Methods

### Study design

**Figure 1:**
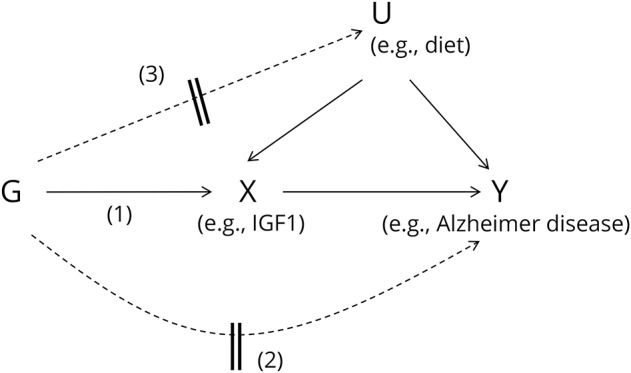
Directed acyclic graph illustrates the mendelian randomization approach Observational studies may have established an association between an exposure (X), such as variation in insulin-like growth factors (IGFs), and outcome (Y), such as risk of Alzheimer disease. These studies will be biased from confounding (U) of the X–Y association that is unmeasured/uncontrolled by statistical models, and possibly other sources of bias such as reverse causation. Mendelian randomization can help to assess whether the exposure is causally related to outcome by using a genetic variant (G) (or several in combination) as an instrumental variable for an exposure. This assumes that the genotypes are robust determinants of the exposure (pathway 1). Due to the independent assortment of alleles for variants between parents and offspring at conception, genotypes that determine the exposure should not also determine confounding factors, nor should disease status modify the genotype (reverse causation).^13^ Therefore, G–Y associations should help to infer a causal relationship of X with Y if instrumental variable assumptions hold. There are potential violations to the framework that can induce direct association of genotypes with outcome independently of the exposure and confounders (pathway 2), or indirectly via confounders (pathway 3). For example, these could arise from horizontal pleiotropy (variants having multiple effects that are independent of exposure determination), linkage disequilibrium between the instrumenting variants and others that affect other traits, or population stratification leading to clustering of variant genotypes and confounding traits.

We conducted a mendelian randomization (MR) analysis (figure 1)^13^ using a 2-sample design: first identifying genetic variants that affect circulating IGF concentrations from published genetic datasets, and then assessing genotype–outcome associations for each identified genetic variant in secondary AD case–control datasets.^14^

### Genetic variant selection

To identify variants as genetic instruments for IGF exposure, we used information from the largest genome-wide association study (GWAS) of circulating IGF1 and IGFBP3 to date.^15^ This identified single nucleotide polymorphisms (SNPs) at 10 independent loci below the threshold for genome-wide significance (*p* values <5 × 10^−8^). Analyses of IGF1 combined data on up to 30,884 individuals (53.3% female) from 21 cohort studies. Analyses of IGFBP3 combined data on up to 18,995 individuals (57.6% female) from 13 cohorts. All participants were of European ancestry. Mean ages within cohorts ranged from 18.9 to 76 years. The published GWAS contains full information on the consortium's studies, participants, genotyping, and IGF assays.^15^ Variants at 7 of the loci determine IGF1, and 4 determine IGFBP3; hits at 2 loci affect both traits. Most of the loci have known or plausible biological links to IGF axis activity.^15,16^ A 10th locus had been found to determine both traits in opposite directions in a bivariate analysis. Hence, the top SNP at this locus was omitted from the main analysis due to potentially conflicting pleiotropic effects on exposures of interest, leaving 9 variants to use, but the additional variant was included in a sensitivity analysis (described below).

### Primary AD case–control sample

Our primary sample for examining genotype–outcome associations consisted of 17,008 late-onset AD cases and 37,154 controls of European ancestry included in the stage 1 GWAS meta-analysis conducted by the International Genomics of Alzheimer's Project (IGAP).^17^ IGAP has published summary statistics of genotype–AD associations for 7,055,881 SNPs online (web.pasteur-lille.fr/en/recherche/u744/igap/igap_download.php). Cases within the consortium's cohorts had mean ages at onset ranging from 68.5 to 82.3 years, of which approximately 60% were women. More details on the stage 1 studies, participants, genotype data, AD diagnostic criteria, and statistical models are described in the published GWAS.^17^

### Replication AD case–control sample

To test for replication of any SNP-AD findings of note, we derived secondary samples of AD cases and controls with genome-wide data available from 4 substudies of individuals in the Swedish Twin Registry (STR).^18–21^ Appendix e-1 (links.lww.com/WNL/A60) contains a detailed description of the sample. Its derivation is depicted in figure e-1 (links.lww.com/WNL/A58), and descriptive characteristics are shown in table e-1 (links.lww.com/WNL/A59). In total, the samples consisted of 984 cases and 10,304 controls.

### Sample overlap

We attempted to quantify the degree of overlap between participants included in the GWAS consortia for IGFs and AD (table e-2, links.lww.com/WNL/A59), which could bias 2-sample MR results if substantial.^22^ The precise degree of overlap could not be determined, but of 19 cohorts included in the main IGF GWAS,^15^ 4 were also part of the IGAP consortium. Up to 10,657 of 30,884 participants (34.5%) included in the GWAS of IGF1 may have been in the IGAP case–control sample (mainly as controls), and up to 8,228 of 18,995 (43.3%) included in the GWAS of IGFBP3. True proportions were probably smaller. Risk of bias from sample overlap is therefore likely to be low.

### Statistical analysis

We based our main analysis on genotype–AD data within AD case–control samples, using IGAP's logistic regression models with genotype as the independent variable, and age, sex, and principal components as covariates. These analyses do not estimate the magnitude of AD risk change per unit difference in the exposure, as other MR models do,^14^ but still help to infer causality and direction of any effects of exposures and outcomes and also benefit from less stringent model assumptions.^23^ For the 9 SNPs of relevance, we extracted β coefficients, standard errors, and the coded effect alleles for logistic regression results of SNP-AD effects from the IGAP meta-analysis. We recoded 5 of the betas by subtracting log-odds values from zero, so that all 9 SNP-AD results were consistently expressed as odds ratios (ORs) and 95% confidence intervals (CIs) according to IGF-raising allele counts of the variants (table 1 for coding and annotation of SNP locations from the dbSNP database; ncbi.nlm.nih.gov/SNP/). We assumed additive effects of allele copies on IGFs. Individual SNP-AD results were combined in a fixed-effects meta-analysis using inverse-variance weighting (IVW)—the overall estimate offering more precision for evidence of causality of IGF effects on AD risk.

**Table 1:**
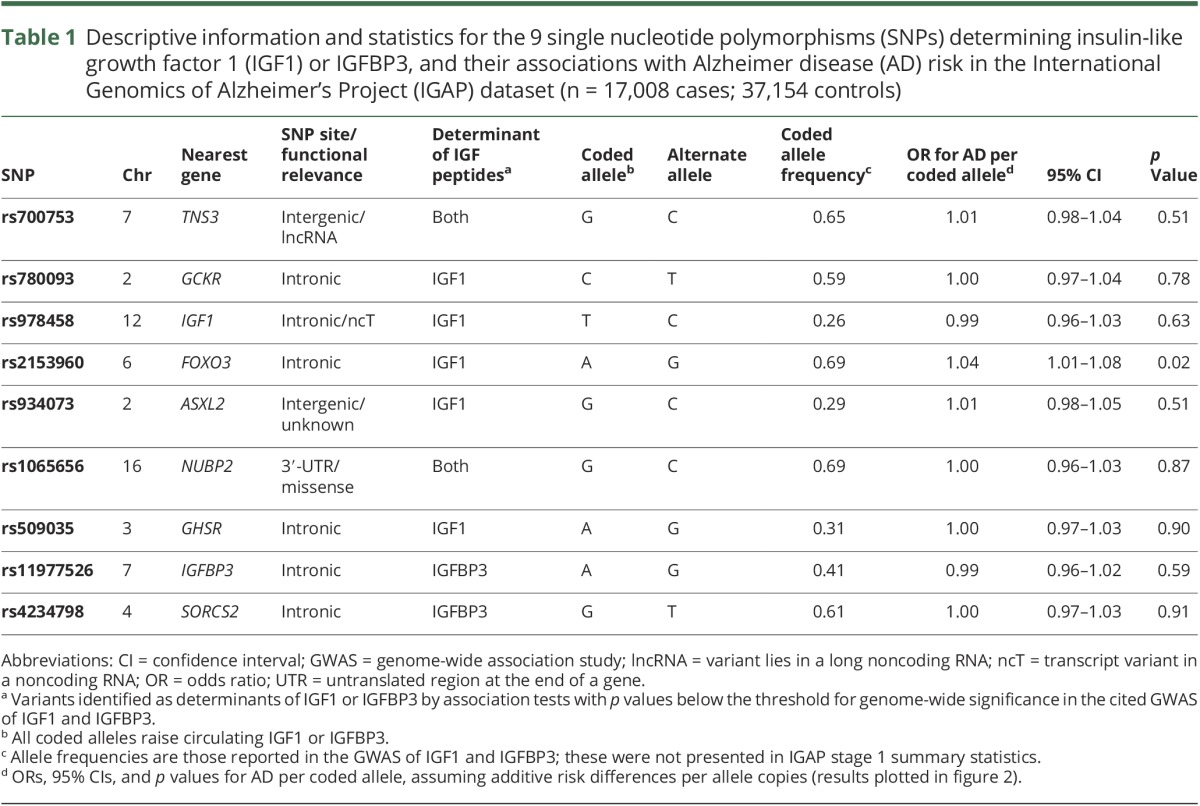
Descriptive information and statistics for the 9 single nucleotide polymorphisms (SNPs) determining insulin-like growth factor 1 (IGF1) or IGFBP3, and their associations with Alzheimer disease (AD) risk in the International Genomics of Alzheimer's Project (IGAP) dataset (n = 17,008 cases; 37,154 controls)

### Sensitivity analyses

Circulating IGF binding proteins may have metabolic effects that are independent of their role in transporting the 2 main IGF ligands.^24^ Thus, we conducted separate analyses to examine whether each genetically instrumented IGF peptide may affect AD risk, with a meta-analysis combining results for the set of 5 SNPs solely affecting total circulating IGF1, and a second meta-analysis combining results for 2 SNPs solely affecting IGFBP3.^15^

The molar ratio of IGF1 to IGFBP3 in circulation may be a better measure of the bioavailability of free IGF1 than separate measures of either component,^25^ and this ratio has been suggested to affect AD risk (where constituent measures have not) in some studies.^9^ Therefore, we conducted a third sensitivity analysis that coded variants according to genotypes expected to increase the ratio of IGF1 to IGFBP3. This meta-analyzed 5 SNP-AD results coding on alleles that raise IGF1 but do not affect IGFBP3 at genome-wide significance, alongside 2 SNPs coded on alleles that lower IGFBP3 but appear not to affect IGF1. In this analysis, we also included an additional variant (rs646776) that appears to have inverse effects on IGF1 and IGFBP3, coding on the allele leading to higher IGF1 and lower IGFBP3.^15^

### Estimating the magnitude of IGF-AD effects

MR estimates of effect magnitudes using the ratio of coefficient method (or others) were precluded for the main analysis because the requisite β coefficients and standard errors for the 9 SNP-IGF results were not reported in the published GWAS, nor available online (results were only available as weighted *z* scores).^15,26^ However, using summary statistics published in another previous GWAS,^16^ a subset of 7 variants were applied for this purpose in an additional analysis of IGF1 (with 3 variants) and IGFBP3 (with 4 variants). We used one set of study-level βs and standard errors of SNP-IGF results from the Framingham Heart Study sample. SNP-IGF results were scaled consistently by SD increases in the peptides per copy of the coded alleles. We calculated SDs from the study interquartile ranges (IQRs) for the IGF peptides, assuming approximately normal distributions of the variables (in which an SD = IQR/1.35 in large samples).^27^ Wald estimators were calculated by dividing the SNP-AD result βs by the SNP-IGF result βs, and standard errors were derived by the delta method.^14^ We then combined Wald estimators for each SNP-AD result in fixed-effects meta-analyses with IVW, giving overall estimates of AD risk difference per SD increases in genetically predicted IGF1 and IGFBP3.

### Testing for violations of MR assumptions

The instrumental variable assumptions made for our models can be violated in several ways (figure 1).^13^ Heterogeneity of SNP-AD results can help to identify variants that may be having pleiotropic effects, and so we reported heterogeneity test statistics from meta-analysis models (Cochran *Q* and *I*^2^).^14^ We considered the use of MR-Egger regression, as a further tool to address pleiotropy.^28^ However, this approach lacks effectiveness when using a small number of variants to instrument exposures, and has lower statistical power for overall effect inference than the IVW method. We did not therefore apply the additional method in this analysis.

### Power calculation

To examine whether we had an effective sample size to undertake the MR analyses, we conducted a power calculation using a published calculator.^29^ This estimated the power for analyses to detect a minimum OR for AD risk per SD difference in IGFBP3 concentration. This used the study-level average *R*^2^ statistic (6.5%) for variance explained in IGFBP3 by the top 4 SNPs determining trait variation in one of the reported GWAS,^16^ along with the sample size (n = 54,162) and proportion of cases (0.314) in the stage 1 IGAP sample. The corresponding *R*^2^ value was not reported for SNP-IGF1 results, so we did not power calculate for the analysis of IGF1.

### Replication analysis

Statistical modeling details for the STR data are provided in appendix e-1 (links.lww.com/WNL/A60).

All analyses were conducted in Stata (version 14.2) and R (version 3.2.2), including the use of R package MR Base.^30^

### Standard protocol approvals, registrations, and patient consents

Written informed consent was obtained from study participants in IGAP and the substudies of the STR or, for those with substantial cognitive impairment within IGAP, from a caregiver, legal guardian, or other proxy instead. IGAP study protocols were reviewed by the local or institutional ethics review boards of the consortium's studies. STR data use was approved by a regional ethics board in Stockholm (DNR 2015/1729-31/5).

## Results

**Figure 2:**
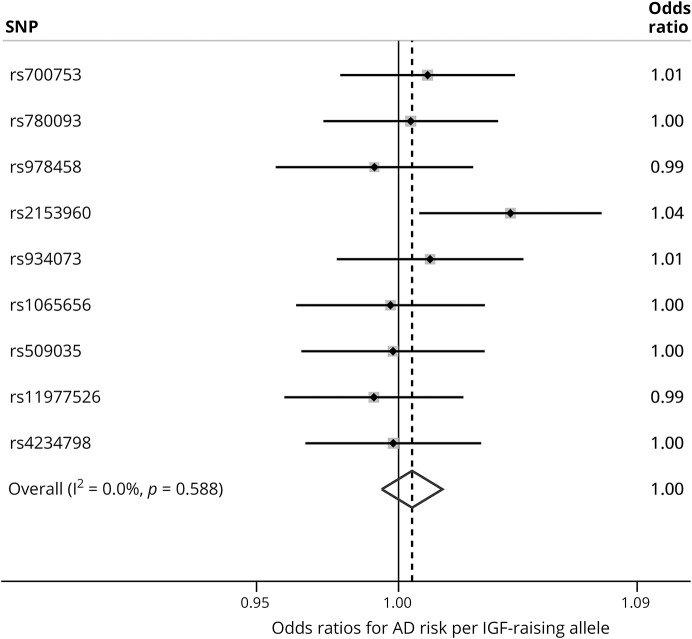
Associations of insulin-like growth factor (IGF)–determining single nucleotide polymorphism (SNP) genotypes with Alzheimer disease (AD) risk, International Genomics of Alzheimer's Project data (n = 17,008 cases; 37,154 controls) A combined estimate and its 95% confidence interval from fixed-effects meta-analysis is shown by the diamond's central position and lateral width, respectively, along with the test statistic of heterogeneity between individual estimates (*I*^2^). Gray boxes around point estimates indicate the weighting of results in the overall estimate.

Table 1 shows statistics for the 9 SNPs used in the main analysis for causal inference. Results of the fixed-effects meta-analysis of the 9 genotype–AD effect estimates are shown in figure 2. The overall estimate indicated no effect of IGF1 or IGFBP3 variation on AD risk. No individual genotype appeared to noticeably affect AD risk, with one exception: rs2153960, which lies in the first intron of the *FOXO3* gene. There was no evidence of heterogeneity between SNP-AD results, meaning that estimates consistently centered across the null. Separate analyses of SNPs solely affecting either IGF1 (figure e-2, links.lww.com/WNL/A58) or IGFBP3 (figure e-3) with AD risk also indicated overall null findings for both sets of variants. Similarly, AD risk did not appear to differ discernibly by differences in genetically instrumented molar ratio of IGF1 to IGFBP3 (figure e-4).

Figures e-5 and e-6 (links.lww.com/WNL/A58) show the estimates of the magnitude of effect of variation in IGF1 and IGFBP3 on AD risk (rather than just inferring causality and effect direction, as in the main analysis). An SD increase in circulating IGF1 would not appear to lead to lower AD risk. There was also no evidence for an effect of an SD increase in IGFBP3 on AD risk. Some heterogeneity was present in the 3 individual IGF1-AD effect estimates—likely driven by the disparity of the result for the *FOXO3* variant rs2153960, compared to the 2 others. No heterogeneity was observed among the 4 IGFBP3-AD effect estimates.

The power calculation for our MR analysis indicated that the sample size should have been effective for identifying an OR of approximately 0.90 or lower per SD increase in IGFBP3 at 80% power, if higher IGFBP3 exposure would be expected to lessen AD risk (or, conversely, an OR ∼1.12 if expected to raise risk).

In the secondary analysis of SNP rs2153960 at the *FOXO3* locus in relation to AD risk, the result was replicated in one sample of STR twins from the TwinGene cohort, but the point estimate was close to the null in the second subsample (figure e-7, links.lww.com/WNL/A58). CIs for both were wide and overlapped with the IGAP estimate—possibly indicating vibration of imprecise estimates around a small effect of the coded allele with AD risk. The overall meta-analysis result for IGAP and STR samples suggested slightly stronger evidence for effect than the IGAP result did alone.

## Discussion

These findings do not support the hypothesis that long-term variation in circulating IGF1 or IGFBP3 cause differences in AD risk. Only 1 of 9 variants that instrumented circulating IGF peptides appeared to affect AD risk, and this may have arisen from pleiotropic effects—i.e., via the influence of the *FOXO3* locus on alternate metabolic pathways, independent of its effects on the IGF axis.

The lack of effect of genetically predicted IGF1 and IGFBP3 on AD risk contrasts many of the conventional case–control studies that reported that disease risk was raised in those with lower circulating measures of IGF peptides.^7,8,11,12,31,32^ Given that measured circulating IGFs are highly responsive to concurrent influences of lifestyle factors that could be affected by disease status, such as recent physical activity, these studies may be particularly prone to bias from reverse causation. Two prospective studies have also investigated whether baseline circulating IGF peptides predict later incident AD risk.^6,10^ Among 3,582 individuals in the US Framingham Heart Study, those grouped in the lowest quartile of baseline IGF1 measures had a higher incidence of AD than participants with higher quartiles (hazard ratio 1.52, 95% CI 1.14–2.00).^6^ In contrast, a study of 745 British men did not find that baseline circulating IGF1, IGFBP3, IGF1:IGFBP3 molar ratio, or IGF2 predicted incident dementia risk (not limited to AD) after 17 years of follow-up, although only 40 dementia cases were recorded.^10^ Given the long prodromal period of AD development (perhaps several decades), reverse causation remains a potential source of bias for prospective studies, including for findings from the Framingham Heart Study samples (mean follow-ups 7 and 8.8 years).^6^ Moreover, residual confounding will have influenced all past observational findings. MR studies are not prone to these typical biases (but come with separate limitations).^13^ Hence, the lack of AD risk difference according to genetically predicted variations in IGF1, IGFBP3, and the molar ratio of these suggests that previous observational evidence implicating these circulating IGF peptides in AD etiology may have been misleading.

It is intriguing to observe a variant within the *FOXO3* locus is linked to AD risk, considering consistent evidence linking FOXO3 to longevity in animal models and human samples.^33,34^ Given the result's incongruence among 8 other null findings for IGF-determining variants, pleiotropic effects may link *FOXO3* function with AD independently of *FOXO3* effects on IGFs. In a GWAS of human longevity (living ≥90 years vs dying sooner), another SNP in *FOXO3*, which is in high LD with rs2153960 (*r*^2^ ∼0.9), ranked highly.^34^ Taking these findings together, the A allele of rs2153960 is linked with both higher risk of AD and a lower chance of reaching the age of 90 years. If variants in the first *FOXO3* intron are truly related to AD via *cis* effects on FOXO3 production, there could be 2 explanations for this link. First, *FOXO3* expression could affect AD development directly, perhaps via effects on apoptosis in neurons or genetic buffering of *APOE* or other loci.^35,36^ Second, *FOXO3* could affect AD risk indirectly via susceptibility to other diseases (i.e., competing risks of death) occurring earlier in the life course.

Strengths of the study include the use of very large AD case–control data, giving sufficient power to detect even small effects. Using multiple variants as instruments for IGF1 and IGFBP3 provided means to identify pleiotropy, which can bias single instrument results in either direction. There are also limitations to this evidence. The SNPs used in these analyses are not ideal instruments for single IGF peptides: several that affect IGF1 also appear to alter IGFBP3 concentrations and vice versa, and perhaps also influence circulating IGF2 (for which we did not find suitable variants to instrument specifically).^37^ Therefore, variants are not indicating AD risk differences attributable to one IGF molecule specifically, although sensitivity analyses using subsets of SNPs that were carefully assessed for magnitudes of effects on each peptide can help in this respect. Moreover, effects of variants on multiple IGF peptides may not invalidate their use as instruments for IGF axis activity as a whole.^37^ We did not address the potential for insulin to influence AD risk; a point of relevance, given that insulin binds to IGF receptors. However, a previous MR study found that AD risk did not differ by genetically predicted variation in insulin.^38^ Population stratification may be another source of bias for MR studies,^13^ but samples were of homogeneous ancestry, and principal components controlling for stratification were included in all analyses. Since the study samples were of white European ancestry, the results are most generalizable to populations of this ethnicity, and less so to others. Finally, MR generally assesses effects of lifelong variation in traits and does not reflect sensitive exposure windows.^13^ This may be important if developmental exposure is critical for later disease risk, and especially if opposite of trait effects in adulthood, e.g., if higher IGF axis activity improved cognitive development, but also conferred risk of sharper cognitive decline in adulthood (or vice versa).

The present results, along with inconsistency across previous conventional epidemiologic studies, suggest that any interventions aimed at increasing circulating IGF1/IGFBP3 to reduce AD risk may fail, and that these traits could be deselected as preventive AD candidates. The findings do not exclude a role of circulating IGF2 or other growth factors in AD development or progression, or of IGFs in other forms of neurodegeneration, and future research could investigate these questions further. Gerontologic and genetic studies should also follow up on how *FOXO3* and its role in aging may intersect with AD etiology.

## Glossary

AD: Alzheimer disease
CI: confidence interval
GWAS: genome-wide association study
IGAP: International Genomics of Alzheimer's Project
IGF: insulin-like growth factor
IQR: interquartile range
IVW: inverse-variance weighting
MR: mendelian randomization
OR: odds ratio
SNP: single nucleotide polymorphism
STR: Swedish Twin Registry
